# Variability in iron, zinc and phytic acid content in a worldwide collection of commercial durum wheat cultivars and the effect of reduced irrigation on these traits

**DOI:** 10.1016/j.foodchem.2017.05.110

**Published:** 2017-12-15

**Authors:** Ana María Magallanes-López, Nayeli Hernandez-Espinosa, Govindan Velu, Gabriel Posadas-Romano, Virginia María Guadalupe Ordoñez-Villegas, José Crossa, Karim Ammar, Carlos Guzmán

**Affiliations:** Global Wheat Program, International Maize and Wheat Improvement Center (CIMMYT), Apdo. Postal 6-641, Mexico DF, Mexico

**Keywords:** Durum wheat, Iron, Zinc, Phytic acid, Nutritional quality

## Abstract

Diets very rich in cereals have been associated with micronutrient malnutrition, and the biofortification of them, has been proposed as one of the best approaches to alleviate the problem. Durum wheat is one of the main sources of calories and protein in many developing countries. In this study, 46 durum varieties grown under full and reduced irrigation, were analyzed for micronutrients and phytate content to determine the potential bioavailability of the micronutrients. The variation was 25.7–40.5 mg/kg for iron and of 24.8–48.8 mg/kg for zinc. For phytate determination (0.462–0.952 %), a modified methodology was validated in order to reduce testing costs while speeding up testing time. Variation was detected for phytate:iron and zinc molar ratios (12.1–29.6 and 16.9–23.6, respectively). The results could be useful to generate varieties with appropriate levels of phytate and micronutrients, which can lead to the development of varieties rich in micronutrients to overcome malnutrition.

## Highlights

• Malnutrition is a major challenge worldwide associated with diets rich in cereals.
• Durum wheat is important source of calories and protein in developing countries.
• A modified scale-down method to quantify phytate was validated.
• 46 durum varieties were analyzed for Fe, Zn and phytate (bioavailability) content.
• Variation was detected for Phy:Fe (12.1–29.6) and Phy:Zn (16.9–23.6) molar ratios.

## 1 Introduction

Malnutrition is a major challenge worldwide and the number of chronically undernourished and malnourished people has been rising (FAO, 2016). Almost 30% of the world population suffers from some form of malnutrition and of these, more than 2 billion people suffer from micronutrient deficiencies, of which 52% are pregnant women and 39% are children under five years of age (FAO, 2016). According to the World Health Organization (WHO) (2002), zinc (Zn) deficiency ranks 11th among the 20 most important risk factors contributing to the burden of disease in the world and 5th among the 10 most important factors in developing countries, while iron (Fe) deficiency ranks 6th. Zinc deficiency is responsible for many severe health complications, including impairments relating to physical growth, the immune system and learning abilities, as well as an increased risk of infections, DNA damage and cancer development (Gibson, 2006). On the other hand, Fe deficiency is the most common cause of anemia globally. Anemia affects around 1.6 billion people worldwide, with pre-school children and pregnant women at the greatest risk (McLean, Cogswell, Egli, Wojdyla, & de Benoist, 2009). Low dietary diversity and diets very rich in cereals have been associated with micronutrient malnutrition (Black et al., 2013). These types of diets are commonly observed in populations of low socioeconomic groups in developing countries.

Biofortification of crops, i.e. enhancing micronutrient concentration in the edible part of the crops by plant breeding, has been proposed as one of the most cost effective and environmentally safe approaches to alleviate malnutrition. The Global Wheat Program of the International Maize and Wheat Improvement Center (CIMMYT), works with the HarvestPlus project to develop biofortified wheat varieties with enhanced Zn concentration. So far, the efforts have been focused on bread wheat (*Triticum aestivum* L. ssp. *aestivum*), which nowadays, is the main wheat species cultivated worldwide (around 90% of the total land), particularly in South Asian countries such as Bangladesh, India and Pakistan where Zn deficiency is a major problem.

However, durum wheat (*Triticum turgidum* L. ssp. *durum*) in many other developing countries (particularly in areas with semiarid climates), is one of the main sources of calories and protein. In North Africa, some countries in West Asia, as well as in Ethiopia, durum wheat is used to prepare diverse foods that serve as a staple food for a large part of the population in those countries; additionally it has socio-cultural and religious values. Among the food products are: couscous, which is popular in North Africa and the Middle East and results from the agglomeration of semolina particles; in the case of the Middle East and particularly Turkey, bulgur is a famous food product which results from parboiling, drying and crushing durum wheat grains; different kinds of leavened breads such as the Algerian Khobz Eddar bread and flat breads such as the Ethiopian kitta or the pancakes named injera; different kinds of porridges as the Ethiopian kinche, prepared with crushed kernels, cooked with milk and water and mixed with spiced butter (Van Damme, 2007); and pasta, a product originally from Italy but that is popular worldwide. Some of these products may be prepared using whole grains or whole-meal flours. Due to this importance as a staple food for different populations, in order to understand the nutritional quality of durum wheat, it is necessary to check the feasibility for developing nutrient-dense durum wheat varieties. The studies carried out so far (Cakmak, Pfeiffer, & McClafferty, 2010; Ficco et al., 2009) have shown that the genetic variation for Zn is not high in durum, although more studies, including on diverse germplasm, are necessary.

On the other hand, with biofortification, breeders can achieve the mineral target increment by directly breeding for higher mineral concentration or breeding for increased bioavailability (Cakmak et al., 2010). Phytic acid (myoinositol-1,2,3,4,5,6-hexakisphosphate), as abundant in the aleurone layer as Fe and Zn, affects the bioavailability of minerals because of the possibility of strong chelation between the two (Coudray et al., 2001). A recent study (Eagling, Wawer, Shewry, Zhao, & Fairweather-tait, 2014) showed that the phytate levels had more influence on Fe bioavailability than total Fe content. Other studies have showed also the relationship between phytate and Zn bioavailability (Frontela, Scarino, Ferruzza, Ros, & Martinez, 2009). Therefore, breeding for low phytate content seems to be a reasonable objective to enhance nutritional quality of any crop.

To achieve new information on the genetic variability for mineral and phytate content in durum wheat, a study was undertaken with the following objectives: 1) describe the variability in grain Fe and Zn and phytic acid concentration in a collection of durum wheat cultivars with worldwide commercial importance; 2) estimate the bioavailability of Zn and Fe in durum whole-meal flours; and 3) examine the effect of reduced irrigation and genotype by environment (G × E) interaction effects on the nutritional quality traits.

## 2 Materials and methods

### 2.1 Plant materials

The study was conducted on a collection of 46 durum wheat varieties composed of representative commercial cultivars from main durum wheat growing countries (Electronic Supplementary Table 1). All genotypes were grown in Ciudad Obregon, Sonora (Mexico) during the 2014–2015 cropping season. All the genotypes were sown in November and harvested in May. Plots were arranged in a randomized complete block design with three replicates under two field management conditions: full irrigation (>500 mm) and reduced irrigation (300 mm), the latter to simulate drought stress. Weed, diseases, and insects were well controlled. Nitrogen (N) was applied (pre-planting) at a rate of 50 kg of N/ha and at tillering, 150 additional units of N were applied in the full irrigation management, while in reduced irrigation, only 50 N units were applied. The amount of nitrogen applied was enough to do not consider nitrogen a restricting factor in the study. At maturity, whole plots were harvested and grain yield was calculated.

The meteorology data of the experimental station in Ciudad Obregon was characterized by almost no precipitation during the wheat growing season. Maximum temperatures were between 30 and 35 °C in March and April, the grain filling time for both treatments. According to the general growing stages of durum wheat in Ciudad Obregon, drought stress was continuous from stem elongation to grain ripening in the reduced irrigation trial.

### 2.2 Grain physical parameters

Whole plots were harvested mechanically and grain yield (t/ha) was determined. A sample of 1 kg of grain was kept for quality analysis. A SeedCount digital imaging system (model SC5000, Next Instruments Pty Ltd, New South Wales, Australia) was used to measure thousand kernel weight (TKW) (g) and test weight (TW) (kg/hL), as it can rapidly and accurately analyze samples of wheat grains and determine the grain number and its morphological characteristics based on software and flatbed scanner technology.

Grain protein content (GPRO, 12.5% moisture basis) was obtained by near-infrared spectroscopy (DA 7200 NIR, Perten Instruments, Sweden), validating its calibration with the chemical Kjeldahl method according to the AACC method 46-12 (American Association of Cereal Chemists, 2010).

### 2.3 Zinc, iron and phytic acid determination

Grain iron (FeC, mg/kg) and zinc (ZnC, mg/kg) concentrations were determined by using a bench-top, non-destructive, energy-dispersive X-ray fluorescence spectrometry (EDXRF) instrument (model X-Supreme 8000, Oxford Instruments plc, Abingdon, UK), which has been standardized for high throughput screening of Zn and Fe in whole grain wheat (Paltridge et al., 2012).

For phytic acid determination, a Megazyme (Ireland) kit was used by making some modifications to the protocol (Megazyme, 2016) provided. A 5 g grain sample was milled into whole-meal flour using a UDY Cyclone Sample Mill (UDY Corporation, USA) with a 5 mm (1/4 inch) screen. One gram of the resulting whole-meal flour was digested with 20 mL of HCl (0.66 M) inside 50 mL Falcon tubes, placed in a mixer with oscillatory agitation (42 oscillations/min) overnight (15 h) at room temperature. The day after, 1 mL of the extract was transferred to a 1.5 mL Eppendorf tube and centrifuged at 13,000 r.p.m. for 10 min. Immediately 0.1 mL of the resulting extract supernatant was transferred to a new tube. The solution was neutralized by the addition of 0.3 mL of a 2:1 (NaOH 0.75 M: HCl 0.66 M) mixture. A control blank sample was included with 0.1 mL of HCl 0.66 M. After that, following the official protocol of Megazyme but with smaller amounts, 1.5 mL Eppendorf tubes were prepared as indicated in Table 1 for the enzymatic dephosphorylation reaction to calculate the free and total phosphorus content of the samples. After this, the tubes were incubated in an Eppendorf Thermomixer at 40 °C for ten minutes. During the first minute of this incubation time, the tubes were shaken at 1400 r.p.m. Following this incubation, different solutions, as indicated in Table 1, were added to the tubes for free and total phosphorus determination and incubated at 40 °C for 15 min with shaking during the first minute at the same speed as mentioned above.

**Table 1:** Preparation of enzymatic dephosphorylation reactions for free and total phosphorus determination.

			Free phosphorus (mL)	Total phosphorous (mL)
Distilled water			0.114	0.094
Solution 1 (buffer provided my Megazyme)			0.04	0.04
Neutralized sample extract			0.02	0.02
Suspension 2 (phytase prepared according to Megazyme)				0.02
*Subtotal (mL)*			*0.174*	*0.174*

Shake the tubes and incubate at 40 °C/10 min				
Distilled water			0.004	
Solution 3 (buffer provided by Megazyme)			0.04	0.04
Suspension 4 (phosphatase alkaline provided by Megazyme)				0.004
*Subtotal (mL)*			*0.044*	*0.044*

After the solutions were mixed with the help of a vortex, 0.06 mL of trichloroacetic acid were added to all tubes. The tubes were centrifuged at 13,000 r.p.m. for 10 min and 0.25 mL of supernatant were transferred to a new tube. To this, 0.125 mL of solution A + B (prepared according to Megazyme manual) were added. This was mixed by vortex and incubated in a water bath set at 40 °C for 1 h. The preparation of the phosphorus calibration curve was done according to Megazyme protocol but used a final volume of 2 mL. Finally, 0.11 mL of the reaction solutions were transferred to a 96-well plate and the absorbance at 655 nm of each well was read in an Epoch Microplate Spectrophotometer. Finally, the calculation of phosphorus and phytic acid content was carried out following Megazyme instructions.

### 2.4 Phytic acid:iron and phytic acid:zinc molar ratios

The contents of phytic acid, Fe and Zn, were converted into moles by dividing by their respective molar mass and atomic weight (660.04, 55.85 and 65.4 g mol^−1^, respectively). The molar ratios of phytic acid:iron (Phy:Fe) and phytic acid:zinc (Phy:Zn) were then calculated.

### 2.5 Statistical analysis

Pearson correlation coefficients (r) and statistical significance for each comparison in the entire study were obtained using SAS. Combined analyses of variance (ANOVA) across environments was performed using procedure Proc Anova of the SAS statistical software.

## 3 Results

### 3.1 Scaling-down method for phytic acid determination

**Fig. 1:**
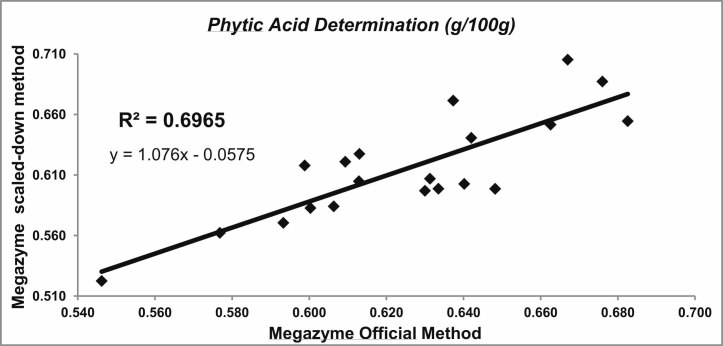
Correlation between phytic acid content obtained using the Megazyme official and scaled-down method in 20 wheat whole-meal samples.

The protocol of the Megazyme kit for phytic acid determination was scaled-down five times and slightly modified to increase the number of samples that can be analyzed with the same amount of reagents and can handle more samples at the same time. The scaled-down method was validated with 20 wheat samples of the breeding program, which were analyzed with both protocols. The analysis was duplicated, with the average of standard deviation for each duplicate of 0.0122 and of 0.0228 for the official Megazyme method and the scaled-down one, respectively. The results obtained (Fig. 1) showed a highly significant correlation between both methodologies (*r* = 0.83). The range of variation for phytic acid content with the Megazyme official method was 0.546–0.683%, with an average value of 0.625%, while for the scaled-down method, the range was 0.522–0.705% with an average value of 0.615%.

### 3.2 Effect of genotype, environment and genotype × environment interaction (G × E) on grain traits

A collection of 46 durum wheat cultivars grown in two different environments was analyzed for diverse grain traits. The combined analysis of variance revealed highly significant effects of the genotype, environment and their interaction (G × E) for all evaluated traits (Table 2). Genotype and environment were the most important factors explaining the variation found followed by G × E. The effect of the environment was particularly high for grain yield (83.2%), phytic acid:Fe (57.8%) and phytic acid (46%). The genotype was the greatest contributor in explaining variation for the rest of traits including FeC and ZnC, except phytic acid:Zn, which was more dependent on the G × E effect (29.8%). G × E was also very important to explain FeC (24.9%).

**Table 2:** Effects of genotype, environment and their interaction (G × E) expressed as % of the total sum of squares from ANOVA analysis.

	DF	Grain yield	TW	TKW	GPRO	FeC	ZnC	Phytic acid	Phy:Fe	Phy:Zn
Genotype	45	10.0	79.6	87.0	66.8	46.3^+^	41.6	42.2	31.0	25.5^+^
Environment	1	83.2	3.3^++^	0.6^□^	22.8	18.3	38.4	46.0^++^	57.8^++^	8.4^□^
G × E	45	2.8	14.2	9.7	7.9	24.9	8.6	7.9	7.0	29.8^++^
Error	180	3.4	2.8	2.2	2.5	10.0	9.1	3.9	4.1	29.7

TW, test weight; TKW, thousand kernel weight; GPRO, grain protein content; FeC, iron content; ZnC, zinc content; Phy:Fe, phytic acid:iron molar ratio; Phy:Zn, phytic acid:zinc molar ratio.

All values were highly significant (p < 0.001), except ^+^significant (p < 0.05); ^++^ (p < 0.01) and ‘□’ not significant.

### 3.3 Kernel characteristics and micronutrients contents

Table 3 shows the means and ranges of the parameters analyzed in the two different environments where the trial was grown under optimum and reduced irrigation conditions. In the reduced irrigation environment, lower grain yield and a higher GPRO than in the full irrigation trial were observed. TKW was slightly higher in full irrigation than in reduced irrigation and the opposite happened for TW. For micronutrients, the FeC was higher in reduced irrigation with a mean of 33.6 mg/kg and a range of 30.2–40.5 mg/kg, while ZnC was higher in the full irrigation environment with a range between 31.8–48.8 mg/kg and a mean of 37.2 mg/kg, compared to the range of 24.8–44.7 mg/kg and average of 30.9 mg/kg in the reduced irrigation environment. A higher mean value for phytic acid was found in full irrigation, and although maximum levels in the two environments were similar, the reduced irrigation environment had lower minimum values than in the full irrigation.

**Table 3:** Comparison between traits means and ranges for full irrigation and reduced irrigation environments.

	Full Irrigation		Reduced Irrigation	
	Mean	Range	Mean	Range
Grain yield (t/ha)	5.1	2.8–6.1	2.6	1.1–3.2
TW (kg/hL)	81.6	74.5–84.0	82.2	78.9–84.4
TKW (g)	45.2	31.6–57.7	44.3	34.1–53.3
GPRO (%)	13.0	11.3–15.8	14.2	12.8–18.2
FeC (mg/kg)	31.3	25.7–39.1	33.6	30.2–40.5
ZnC (mg/kg)	37.2	31.8–48.8	30.9	24.8–44.7
Phytic acid (%)	0.747	0.654–0.945	0.604	0.483–0.919
Phy:Fe	20.4	16.3–29.6	15.2	12.1–20.4
Phy:Zn	20.2	17.4–23.6	19.2	16.9–22.1

TW, test weight; TKW, thousand kernel weight; GPRO, grain protein content; FeC, iron content; ZnC, zinc content; Phy:Fe, phytic acid:iron molar ratio; Phy:Zn, phytic acid:zinc molar ratio.

Variation for micronutrients and phytic acid was also detected among cultivars (Electronic Supplementary Table 1). In the full irrigation environment cv. *Don Jaime* (39.1 mg/kg) and cvs. *Iride* and *Rafi C97* (25.7 and 26.3 mg/kg, respectively) showed the highest and lowest values for FeC, respectively. The largest ZnC was presented in cv. *Normanno* (48.8 mg/kg) and the lowest content in cv. *Rafi C97* (31.8 mg/kg). Highest phytic acid contents were obtained in cvs. *Exeldur* and *Normanno* (0.94 and 0.88%, respectively) and the lowest values were presented in cv. *Altar 84* and cv. *Malavika* (0.66 and 0.65%, respectively).

With respect to reduced irrigated environment, the highest content of FeC was obtained by cv. *Bellaroi* (40.5 mg/kg) and the lowest concentrations corresponded with cvs. *Calero* and *Tomouh* (30.3 and 30.2 mg/kg, respectively). Cvs. *Exeldur* and *Bellaroi* had the highest ZnC (44.7 and 43.0 mg/kg, respectively), while cvs. *Altar 84* and *Nasr 99* varieties showed the lowest contents (24.8 and 25.7 mg/kg, respectively). For phytic acid, the highest concentration was obtained by the variety *Bellaroi* (0.92%) and the lowest content was found in cvs. *Calero* and *Don Jaime* (0.49 and 0.48%, respectively).

Significant variation was also found among cultivars (Electronic Supplementary Table 1) and between environments (Table 3) for phytic acid:micronutrients (Fe and Zn) molar ratios. For Phy:Fe and Zn molar ratios, the reduced irrigation environment showed lower values compared to the full irrigation one, although this difference was much more important for Fe than for Zn. The cvs. *Don Jaime* (16.3) and *Duilio* (17.4) showed the lowest Phy:Fe and Zn molar ratios, respectively, in the full irrigation environment, while the cvs. *Don Jaime* (12.1) and *Nacori C97* (16.9) had the lowest Phy:Fe and Zn molar ratios, respectively, in the reduced irrigation environment.

### 3.4 Correlations between micronutrients content, phytic acid and kernel characteristics

To analyze the relationships among microelement concentrations and phytic acid with kernel characteristics, the Pearson correlation analysis was conducted (Table 4). Several traits showed consistent correlations between them across both environments. For example, the correlations between GPRO and both micronutrients, and between GPRO and phytic acid were highly significant in both environments but stronger in the reduced irrigation environment. Another highly significant correlation in both environments was between ZnC and phytic acid. The correlation between FeC and phytic acid was also highly significant in the reduced irrigation environment but not significant in the full irrigation one. These general associations were found in specific cultivars. For example, cv. *Normanno* had a relatively high FeC (35.0 mg/kg) and GPRO (15.8%) and the highest values recorded for ZnC (48.8 mg/kg) and phytic acid (0.88%) in the full irrigation environment.

**Table 4:** Pearson correlation coefficients (*r*) among micronutrients grain components and grain yield data obtained in two environments.

	Grain yield		TW	TKW	GPRO	FeC	ZnC
*Full irrigation*							
Grain yield	1						
TW	0.58^**^		1				
TKW	0.24^*^		0.46^**^	1			
GPRO	−0.39^**^		−0.47^**^	0.05	1		
FeC	0.17		0.11	0.47^**^	0.29^**^	1	
ZnC	−0.29^**^		−0.41^**^	0.10	0.67^**^	0.35^**^	1
Phytic Acid	−0.55^**^		−0.62^**^	−0.10	0.73^**^	0.09	0.71^**^

*Reduced irrigation*							
Grain yield	1						
TW	0.46^**^	1					
TKW	0.20	0.25*		1			
GPRO	−0.60^**^	−0.49^**^		−0.03	1		
FeC	−0.34^**^	0.14		0.17	0.65^**^	1	
ZnC	−0.52^**^	−0.41^**^		−0.03	0.73^**^	0.62^**^	1
Phytic Acid	−0.39^**^	−0.38^**^		0.0	0.80^**^	0.64^**^	0.77^**^

TW, test weight; TKW, thousand kernel weight; GPRO, grain protein content; FeC, iron content; ZnC, zinc content.

^*^^,^^**^ significant at 5% and 1% probability levels, respectively.

Grain density (TW) was also negatively correlated with ZnC in both environments but not with FeC. Similarly, TKW was not correlated with FeC in the reduced irrigation environment and with ZnC in both the environments. Grain yield also showed significant negative correlations with several traits including FeC (only significant in the reduced irrigation environment), ZnC and phytic acid. Using the same previous example, cv. *Normanno* had the highest values for ZnC (48.8 mg/kg) and grain yield slightly below average (4.8 t/ha) in the full irrigation environment. In the case of phytic acid, the Pearson coefficient was -0.55 with grain yield in full irrigation. The cv. *Exeldur* showed the highest phytic acid content (0.94%) and the lowest value of grain yield (2.8 t/ha). For reduced irrigation environment, lower grain yield was significantly associated with an increase in FeC (*r* = −0.34), ZnC (*r* = −0.52) and phytic acid (*r* = −0.39). An example of this is presented by cvs. *Normanno* and *Bellaroi*, which had high values of FeC (38.5 and 40.5 mg/kg, respectively), ZnC (37.2 and 43.0 mg/kg, respectively) and phytic acid (0.79 and 0.92%, respectively) but with low grain yields (1.8 and 1.5 t/ha, respectively).

The correlations between phytic acid:micronutrients molar ratios and other grain traits were also analyzed (Table 5). In most cases, there was a negative correlation between grain density (TW) and Phy:Fe or Zn molar ratio. That meant that in most cases the better the grain soundness, the higher the potential bioavailability (less phytic acid for more micronutrients). In full irrigation, the cv. *Exeldur* presented the highest Phy:Fe value (29.6) and the lowest grain density (TW = 74.5 kg/hL), whereas for reduced irrigation, cv. *Bellaroi* had the lowest value for TW (79.9 kg/hL) and high molar ratios for both micronutrients (19.7 for Phy:Fe and 21.9 for Phy:Zn).

**Table 5:** Association between Molar Ratio, TW, TKW, GPRO and GY for full irrigation and reduce irrigation traits.

	Full irrigation		Reduced irrigation	
	Phy:Fe	Phy:Zn	Phy:Fe	Phy:Zn
Grain yield	−0.57^**^	−0.23^*^	−0.31^**^	0.01
TW	−0.55^**^	−0.09	−0.42^**^	−0.06
TKW	−0.44^**^	−0.22^*^	−0.08	0.02
GPRO	0.31^**^	−0.08	0.65^**^	0.17

TW, test weight; TKW, thousand kernel weight; GPRO, grain protein content; FeC, iron content; ZnC, zinc content; Phy:Fe, phytic acid:iron molar ratio; Phy:Zn, phytic acid:zinc molar ratio.

^*^^,^^**^ significant at 5% and 1% probability levels, respectively.

Significant relationship was also detected between GPRO and Phy:Fe, but not with Phy:Zn. Cvs. *Bellaroi*, *Exeldur* and *Normanno* showed the highest content of GPRO (15.4, 15.4 and 15.8%, respectively) and high Phy:Fe (31.7, 28.1 and 35.0, respectively); for reduced irrigation the same varieties presented high Phy:Fe (19.7, 20.4 and 17.2, respectively) and high values of GPRO (18.2, 17.3 and 18.0%, respectively). In contrast, grain yield showed negative correlations with Phy:Fe in both environments and with Phy:Zn in the full irrigation environment. In full irrigation, cv. *Exeldur* had the lowest grain yield (2.8 t/ha) but corresponded with the highest value of Phy:Fe (29.6); and for reduced irrigation environment cvs. *Exeldur*, *Normanno* and *Bellaroi* varieties presented the highest values for Phy:Zn (20.4, 17.2 and 19.7, respectively) with the lowest grain yields (1.1, 1.8 and 1.5 t/ha, respectively).

## 4 Discussion

So far, wheat biofortification breeding efforts for micronutrients (Zn and Fe) have been mainly focused on bread wheat, which have led to the release of several varieties in target countries (India and Pakistan) led by the consortium of the HarvestPlus challenge program. These biofortified varieties have shown competitive grain yields and approximately 30–40% more ZnC compared with the conventional varieties grown in those areas (Velu et al., 2015). This achievement was possible due to the different wheat genetic resources with high ZnC preserved at CIMMYT’s germplasm bank (Guzmán et al., 2014) and were crossed with modern elite wheat lines, which are not very variable for micronutrients content (Cakmak et al., 2010).

To carry out a similar breeding process with durum wheat, it is necessary to know the current baseline micronutrient levels in commercial cultivars and the magnitude of genetic variability available within the primary genepool. It is also important to have high-throughput methodologies that allow for the fast analysis of hundreds of samples generated by the breeding program at a low cost. The EDXRF (energy-dispersive X-ray fluorescence spectrometry) equipment (Paltridge et al., 2012) has been an extremely useful tool for analyzing ZnC and FeC and was used in this study for the analysis of grain samples from 46 commercial durum wheat varieties with worldwide economic importance, grown under full and reduced irrigation (simulated drought stress). The range of variation found in this worldwide collection across the whole trial was of 25.7–40.5 mg/kg for FeC and of 24.8–48.8 mg/kg for ZnC. In the case of ZnC, this range is similar to that found by Ficco et al. (2009) (28.5–46.3 mg/kg) in a set of modern durum cvs. from Italy, although their range for FeC (33.6–65.6 mg/kg) was higher than that found in the current study. The study of Ficco et al. (2009) is the only reported study which was carried out under field conditions with a significant number of modern durum cultivars. Other authors have found similar or smaller ranges of variation in studies done with a small number of genotypes and/or under greenhouse conditions (Cakmak et al., 2000, Genc and McDonald, 2008; Hakki et al., 2014; Rachoń, Pałys, & Szumiło, 2012; Zhao et al., 2009). Therefore, it seems clear that the genetic variability available in the modern durum pool is not enough, and it would be necessary to use other wheat genetic resources in the breeding process. In this respect, Cakmak et al. (2000) and Cakmak et al. (2004) and Gomez-Becerra et al. (2010) have shown that *T. dicoccoides* could be a good source of high micronutrients concentration along with *T. dicoccum* (Monasterio & Graham, 2000).

To increase micronutrients intake from wheat, it is not only the concentration of the micronutrient that is important, but also the amount that is available for absorption (Frontela et al., 2009). Phytic acid, an abundant component of the wheat grain that serves as phosphorus reservoir, is also considered to be an anti-nutrient because it chelates Fe and Zn during the digestion and avoids their absorption. In fact, phytic acid:micronutrients molar ratios are used to estimate the potential bioavailability of the micronutrients. In general terms, there is higher mineral bioavailability when the molar ratio is low and *vice versa*. For Phy:Fe, the molar ratio should be <1 or preferably <0.4 to significantly improve Fe absorption (Hurrel & Egli, 2010), while for Phy:Zn molar ratios <5, between 5 and 15 and >15 have been associated with high, moderate and low zinc bioavailability, corresponding to approximately 50%, 30% and 15% of total zinc, respectively (Gibson, 2006). Because of this, the variability for phytic acid was also examined in the current study.

For this purpose, a modified methodology to quantify phytic acid was validated. While different methods to determine the amount of phytic acid in wheat have been described (Dost and Tokul, 2006, Haug and Lantzsch, 1983), the costs are high and the methods are designed to evaluate only limited number of samples per day. In breeding programs, the analysis of a large number of samples has to be done in the shortest time possible with the lowest costs. Due to this, we worked in the modification of the simple and accurate commercial kit of Megazyme to determine phytic acid, which was scaled-down in order to reduce testing costs while speeding up testing time. The modifications done were allowed to use smaller disposable components (1.5–2.0 ml Eppendorf tubes) and more efficient equipment for the different steps of the protocol (Thermomixer and Centrifuges for Eppendorf tubes and spectrophotometer for 96 well plates). This implies handling a higher number of samples per day (up to 50 with one technician), significantly reducing the cost of the analysis (5 times), and keeping enough accuracy to make selection in a breeding program (high correlation with the conventional method, *r* = 0.83). The use of a commercial-kit with worldwide distribution (Megazyme) could facilitate the implementation of the described method in other wheat quality labs working for the same (HarvestPlus) or similar projects, making the extrapolation of results found among breeding programs much easier.

The variation found for phytic acid ranged from 0.462 to 0.952% (2-fold variation) in the whole trial, with an average of 0.675%. Branković et al. (2015) reported a smaller range of variation for phytic acid but with significantly higher values (1.463–1.678%) in a set of 15 durum genotypes (nine with CIMMYT origin), which shows the importance of the environmental conditions and methodology used when dealing with this trait. Tabekha and Donnelly (1982) also found higher values in six durum cvs. from USA grown in three locations (0.95–1.43%, average of 1.09%). However, Tavajjoh, Yasrebi, Karimian, and Olama (2011) found more similar values to the ones of the current study in two Iranian durum cvs. (0.879 and 0.740%), as well as Hussain, Maqsood, and Miller (2012) in 65 bread wheat varieties grown in Pakistan (0.706–1.113%).

Besides the Phy:Fe and Phy:Zn molar ratios were calculated and an interesting variation was detected (12.1–29.6 and 16.9–23.6, respectively) in the current set of data. This means that there was an almost two fold variation for Phy:Zn and around 1.5-fold for Phy:Fe, although all the varieties fall in the category of low bioavailability for both Fe and Zn according to Gibson (2006) and Hurrel and Egli (2010). The literature about durum wheat grain Phy:Fe is scarce or nonexistent. Salunke et al. (2014) showed findings very similar to this study, with a range Phy:Fe of 15.5–31.3 in a set of nine bread wheat varieties. Eagling et al. (2014) reported Phy:Fe around twelve in two bread wheat whole-meal flour samples, while Akhter, Saeed, Irfan, and Malik (2012) gave a range of 1.96–3.86 for the same trait in white flour of twelve bread wheat varieties. For Phy:Zn there is more information available, with Hussain et al. (2012) reporting a range of 23.9–41.4 for durum wheat, Erdal et al. (2002) reporting 49–116 in durum and 29–178 in bread wheat, and Tavajjoh et al. (2011) reporting 26.5 and 26.9 in two durum cvs., which were in agreement with our data. Therefore, the concentration of micronutrients and the molar ratios revealed by the current and previous studies are not adequate to meet the daily requirements of humans in countries where durum wheat represents the main source of calories. Durum breeding programs working for target areas where micronutrient deficiency is a problem should be more focused on improving micronutrient concentration and reducing phytic acid to alleviate the malnutrition problems of the region.

This study revealed more information which will be useful for devising an appropriate durum wheat breeding strategy focused on improving nutritional quality. Although the genetic variability found was not very large, the genetic control of most of the traits seems to be high, which probably results in much faster genetic gains through proper selection methods. This would be more difficult to obtain for FeC and Phy:Zn due to the considerable G × E effect on those traits. Ficco et al. (2009) found, in general, a larger environment and, more importantly, G × E effect for those traits, which would significantly slow-down the genetic progress for these traits if confirmed. Another interesting fact found, previously reported by other authors (Gomez-Becerra et al., 2010, Kutman et al., 2010, Zhao et al., 2009), is that both micronutrient concentrations are correlated with protein content, which in practice means that increasing the nutritional quality of the durum cultivars would also lead to an indirect increase in the industrial quality of the grain. Neither the protein nor the micronutrients and phytic acid concentration were affected by grain size in most of the cases (only FeC in full irrigation environment was affected by TKW), which removes the presence of a dilution or concentration effect of these components due to grain size. This agrees with Ficco et al. (2009) for FeC and ZnC but not for phytate. However, we could speak of a dilution or concentration effect due to changes in the grain density (significant correlations with TW for most of the components) and in the whole plant grain yield, which was negatively associated with all the grain components in both environments. This has been previously reported by several authors (Ficco et al., 2009, Liu et al., 2014, Velu et al., 2016) and would negatively affect the breeding process, where strong selection would need to be applied to break the barrier of the negative association between grain yield and micronutrients. At least this negative association was stronger in most of the cases between grain yield and phytic acid, which means that increasing yield will indirectly contribute to increasing the bioavailability of the micronutrients. This fact was confirmed with the negative correlation found between grain yield and Phy:micronutrients molar ratios, with the exception of Phy:Zn in the reduced irrigation environment.

Lastly, due to drought stress, which is quite frequent in the main durum wheat growing areas (Mediterranean countries and Middle East), it was interesting to observe the effect of water stress on nutritional quality of durum wheat. A greater FeC was found in reduced irrigation, in agreement with Guzmán et al. (2016) in a study done in a similar environment but with smaller number of cultivars. However, in the current study, significantly lower ZnC was found in the reduced irrigation environment, which contradicts previous studies in both durum and bread wheats (Guzmán et al., 2016; Velu et al., 2016). These results are probably because there was not a remarkable difference in grain size (TKW) across environments, and the zinc uptake was severely reduced by the water stress or lesser grain filling period, which might reduce the loading of more Zn in the grain. The Phy:micronutrients molar ratios were also somehow smaller in reduced irrigation, indicating potentially better bioavailability of Fe and Zn when durum is grown under water stress.

## 5 Conclusions

The data generated in the present study has shown differences in micronutrients (Fe and Zn) and phytic acid in a worldwide durum collection, along with the evaluation of the responses to the environments (full and reduced irrigation or drought stress). The results could be useful for breeders to generate varieties with appropriate levels of phytic acid and micronutrients, which can lead to the development of variety-based products rich in the desired minerals to overcome deficiencies in population groups suffering from hidden hunger related issues of micronutrient bioavailability.

## Conflict of interest statements

The authors declare that they do not have any conflict of interest.

## References

[b0005] Akhter S., Saeed A., Irfan M., Malik K.A. (2012). In vitro dephytinization and bioavailability of essential minerals in several wheat varieties. Journal of Cereal Science.

[b0010] American Association of Cereal Chemists (2010). Approved methods of the AACC.

[b0015] Black R.E., Victora C.G., Walker S.P., Bhutta Z.A., Christian P., De Onis M., Uauy R. (2013). Maternal and child undernutrition and overweight in low-income and middle-income countries. Lancet.

[b0020] Branković G., Dragičević V., Dodig D., Knežević D., Kandić V., Šurlan-Momirović G., Sečanski M. (2015). Phytic acid, inorganic phosphorus, antioxidants in bread and durum wheat and their associations with agronomic traits. Agricultural and Food Science.

[b0025] Cakmak I., Ozkan H., Braun H., Welch R., Romheld V. (2000). Zinc and iron concentrations in seeds of wild, primitive, and modern wheats. Food and Nutrition Bulletin.

[b0030] Cakmak I., Pfeiffer W., McClafferty B. (2010). Biofortification of durum wheat with zinc and iron. Cereal Chemistry.

[b0035] Cakmak I., Torun A., Millet E., Feldman M., Fahima T., Korol A., Ozkan H. (2004). Triticum dicoccoides: An important genetic resource for increasing Zinc and Iron concentration in modern cultivated wheat. Japanese Society of Soil Science and Plant Nutrition.

[b0040] Coudray C., Levrat-Verny M.A., Tressol J.C., Feillet-Coudray C., Horcajada-Molteni N.M., Demigné C., Rémésy C. (2001). Mineral supplementation of white wheat flour is necessary to maintain adequate mineral status and bone characteristics in rats. Journal of Trace Elements in Medicine and Biology.

[b0045] Dost K., Tokul O. (2006). Determination of phytic acid in wheat and wheat products by reverse phase high performance liquid chromatography. Analytica Chimica Acta.

[b0050] Eagling T., Wawer A.A., Shewry P.R., Zhao F., Fairweather-tait S.J. (2014). Iron bioavailability in two commercial cultivars of wheat: Comparison between wholegrain and white flour and the effects of nicotianamine and 2′-deoxymugineic acid on iron uptake into Caco – 2 cells. Journal of Agricultural and Food Chemistry.

[b0055] Erdal I., Yilmaz A., Taban S., Eker S., Torun B., Cakmak I. (2002). Phytic acid and phosphorus concentrations in seeds of wheat cultivars grown with and without zinc fertilization. Journal of Plant Nutrition.

[b0060] FAO (2016). El espectro de la malnutricion. www.fao.org/worldfoodsummit/spanish/fsheets/malnutrition.pdf. Accessed 18.01.17.

[b0065] Ficco D.B.M., Riefolo C., Nicastro G., De Simone V., Di Gesù A.M., Beleggia R., De Vita P. (2009). Phytate and mineral elements concentration in a collection of Italian durum wheat cultivars. Field Crops Research.

[b0070] Frontela C., Scarino M.L., Ferruzza S., Ros G., Martinez C. (2009). Effect of dephytinization on bioavailability of iron, calcium and zinc from infant cereals assessed in the Caco-2 cell model. World Journal of Gastroenterology.

[b0075] Genc Y., McDonald G.K. (2008). Domesticated emmer wheat [*T. turgidum* L. subsp. dicoccon (Schrank) Thell.] as a source for improvement of zinc efficiency in durum wheat. Plant and Soil.

[b0080] Gibson R.S. (2006). Zinc: the missing link in combating micronutrient malnutrition in developing countries. Proceedings of the Nutrition Society.

[b0085] Gomez-Becerra H.F., Yazici A., Ozturk L., Budak H., Peleg Z., Morgounov A., Cakmak I. (2010). Genetic variation and environmental stability of grain mineral nutrient concentrations in Triticum dicoccoides under five environments. Euphytica.

[b0090] Guzmán C., Autrique J.E., Mondal S., Singh R.P., Govindan V., Morales-Dorantes A., Peña R.J. (2016). Response to drought and heat stress on wheat quality, with special emphasis on bread-making quality, in durum wheat. Field Crops Research.

[b0095] Guzmán C., Medina-Larqué A.S., Velu G., González-Santoyo H., Singh R.P., Huerta-Espino J., Peña R.J. (2014). Use of wheat genetic resources to develop biofortified wheat with enhanced grain zinc and iron concentrations and desirable processing quality. Journal of Cereal Science.

[b0100] Hakki E.E., Dograr N., Pandey A., Khan M.K., Hamurcu M., Kayis S.A., Akkaya M.S. (2014). Molecular and elemental characterization of selected Turkish durum wheat varieties. Notulae Botanicae Horti Agrobotanici Cluj-Napoca.

[b0105] Haug W., Lantzsch H.J. (1983). Sensitive method for the rapid determination of phytate in cereals and cereals products. Journal of the Science Food and Agriculture.

[b0110] Hurrell R., Egli I. (2010). Iron bioavailability and dietary reference values. The American Journal of Clinical Nutrition.

[b0115] Hussain S., Maqsood M.A., Miller L.V. (2012). Bioavailable zinc in grains of bread wheat varieties of Pakistan. Cereal Research Communications.

[b0120] Kutman U.B., Yildiz B., Ozturk L., Cakmak I. (2010). Biofortification of durum wheat with zinc through soil and foliar applications of nitrogen. Cereal Chemistry.

[b0125] Liu H., Wang Z.H., Li F., Li K., Yang N., Yang Y., Qiu W. (2014). Grain iron and zinc concentrations of wheat and their relationships to yield in major wheat production areas in China. Field Crops Research.

[b0130] McLean E., Cogswell M., Egli I., Wojdyla D., de Benoist B. (2009). Worldwide prevalence of anaemia, WHO Vitamin and Mineral Nutrition Information System, 1993–2005. Public Health Nutrition.

[b0135] Megazyme (2016). https://secure.megazyme.com/files/Booklet/K-PHYT_DATA.pdf. Accessed 13.02.17.

[b0140] Monasterio I., Graham R. (2000). Breeding for trace minerals in wheat. Food and Nutrition Bulletin.

[b0145] Paltridge N.G., Milham P.J., Ortiz-Monasterio J.I., Velu G., Yasmin Z., Palmer L.J., Stangoulis J.C.R. (2012). Energy-dispersive X-ray fluorescence spectrometry as a tool for zinc, iron and selenium analysis in whole grain wheat. Plant and Soil.

[b0150] Rachoń L., Pałys E., Szumiło G. (2012). Comparison of the chemical composition of spring durum wheat grain (*Triticum durum*) and common wheat grain (*Triticum aestivum* ssp. Vulgare). Journal of Elementology.

[b0155] Salunke R., Rawat N., Neelam K., Tiwari V.K., Randhawa G.S., Dhaliwal H.S., Roy P. (2014). Effect of grain hardness on bioavailability of iron in wheat as determined using the coupled invitro digestion/Caco-2 model. LWT – Journal of Food Science and Technology.

[b0160] Tabekha M.M., Donnelly B.J. (1982). Phytic acid in durum wheat and its milled products. Cereal Chemistry.

[b0165] Tavajjoh M., Yasrebi J., Karimian N., Olama V. (2011). Phytic acid concentration and phytic acid: Zinc molar ratio in wheat cultivars and bread flours, Fars province, Iran. Journal of Agricultural Science and Technology.

[b0170] Van Damme P. (2007). Plant resources of tropical Africa 1. Cereals and pulses. Economy Botany.

[b0175] Velu G., Guzman C., Mondal S., Autrique J.E., Huerta J., Singh R.P. (2016). Effect of drought and elevated temperature on grain zinc and iron concentrations in CIMMYT spring wheat. Journal of Cereal Science.

[b0180] Velu G., Singh R., Arun B., Mishra V.K., Tiwari C., Joshi A., Pfeiffer W.H. (2015). Reaching out to farmers with high zinc wheat varieties through public-private partnerships – An experience from eastern-gangetic plains of India. Advances in Food Technology and Nutrition Sciences.

[b0185] World Health Organization (WHO). (2002). Ten leading causes of illness and disease in low income countries. http://www.who.int/mediacentre/factsheets/fs310_2008.pdf. The World Health Report 2002 Geneva: WHO. Accessed 13.02.17.

[b0190] Zhao F.J., Su Y.H., Dunham S.J., Rakszegi M., Bedo Z., McGrath S.P., Shewry P.R. (2009). Variation in mineral micronutrient concentrations in grain of wheat lines of diverse origin. Journal of Cereal Science.

