# Supplementary material for: Variability in iron, zinc and phytic acid content in a worldwide collection of commercial durum wheat cultivars and the effect of reduced irrigation on these traits
Source: Food Chem. 2017 Dec 15;237:499–505. doi: 10.1016/j.foodchem.2017.05.110 (PMC5544597; doi:10.1016/j.foodchem.2017.05.110)
Supplement: Supplementary Table 1 [file mmc1.pdf]

Electronic Supplementary Table 1. Grain yield and grain quality data of 46 durum wheat cvs. grown under full irrigation (A) and under reduced irrigation (B).

A: Full irrigation

Entry	Country of origin	Cv.	Grain Yield (t/ha)	Test Weight (TW) kg/Hl	Thousand Kernel Weight (TKW) g	Grain Protein (GPRO) 12.5%MB	Iron Content (FeC) mg/Kg	Zinc Content (ZnC) mg/Kg	Phytic Acid (%)	Molar Ratio Phy:Fe	Molar Ratio Phy:Zn
1	ALGERIA	**KHROUB 76**	4,5	80,4	47,4	13,7	28,6	41,4	0,83	24,4	20,4
2	ALGERIA	**WAHA**	5,3	82,4	43,4	13,9	30,1	39,0	0,74	20,7	18,8
3	ARGENTINA	**BONAERENSE VALVERDE**	5,3	83,1	39,1	11,9	27,8	36,1	0,68	21,0	18,8
4	ARGENTINA	**BUCK CANDISUR**	3,8	81,6	53,4	13,7	32,8	40,2	0,83	21,7	20,7
5	AUSTRALIA	**BELLAROI**	4,4	79,8	48,8	15,4	31,7	43,0	0,86	22,8	19,8
6	AUSTRALIA	**HYPERNO**	5,0	81,5	41,1	12,0	30,5	34,8	0,69	18,9	19,7
7	AUSTRALIA	**JANDAROI**	5,2	81,1	48,4	14,2	28,3	38,0	0,76	23,6	20,3
8	CHILE	**GUAYACAN INIA**	5,3	83,2	54,6	12,4	31,7	36,1	0,74	19,6	20,3
9	SPAIN	**AVISPA**	5,1	82,0	41,8	12,5	27,9	33,3	0,75	22,5	22,3
10	FRANCE	**EXELDUR**	2,8	74,5	31,6	15,4	28,1	43,8	0,94	29,6	21,3
11	INDIA	**HD 4530**	5,0	79,9	39,5	12,6	32,0	36,0	0,70	18,6	19,9
12	INDIA	**MALAVIKA**	5,0	82,9	45,8	11,8	30,3	35,6	0,65	18,5	18,4
13	INDIA	**RAJ 1555**	4,9	80,5	49,5	12,0	30,8	33,9	0,71	19,7	20,7
14	INDIA	**WH 896**	5,4	82,8	45,3	12,0	29,5	33,6	0,66	18,5	19,4
15	ITALIA	**BRONTE**	5,5	81,6	52,2	13,4	35,3	39,6	0,71	16,8	18,5
16	ITALIA	**CRECALE**	4,6	79,9	37,0	13,2	28,5	38,9	0,78	23,1	20,1
17	ITALIA	**DUILLIO**	5,9	83,2	50,5	12,8	30,8	39,0	0,69	18,6	17,4
18	ITALIA	**IRIDE**	4,4	81,3	38,9	12,3	25,7	33,7	0,76	25,5	23,6
19	ITALIA	**KARALIS**	4,9	80,9	48,1	13,5	35,3	45,2	0,79	19,1	17,9
20	ITALIA	**NORMANO**	4,8	78,5	41,7	15,8	35,0	48,8	0,88	20,9	17,9
21	ITALIA	**SARAGOLLA**	5,6	82,6	45,3	12,4	32,2	38,1	0,75	19,8	19,7
22	ITALIA	**SVEVO**	5,2	81,9	44,1	14,1	30,9	38,1	0,75	20,4	19,7
23	MEXICO	**ACONCHI 89**	4,8	84,0	46,4	12,4	29,1	37,0	0,75	22,3	21,0
24	MEXICO	**ALTAR 84**	5,3	84,0	44,0	11,9	31,7	34,9	0,66	17,5	19,8
25	MEXICO	**CEMEXI C 2008**	5,8	83,5	42,6	12,6	29,4	34,6	0,70	19,9	20,0

26	MEXICO	**NACORI C 97**	5,9	82,9	44,7	12,9	32,0	32,8	0,67	17,6	20,5
27	MEXICO	**RAFI C 97**	5,4	83,7	37,3	11,7	26,3	31,8	0,68	22,7	22,7
28	MEXICO	**RIO COLORADO**	5,6	79,7	41,3	11,9	31,7	33,1	0,67	18,0	20,4
29	MEXICO	**SOOTY_9/RASCON_37**	5,5	82,5	44,4	11,3	31,6	33,2	0,66	18,1	19,9
30	MOROCCO	**AMRIA**	5,3	82,6	51,6	14,1	32,7	37,4	0,82	21,0	21,9
31	MOROCCO	**MARZAK**	5,5	81,6	47,5	13,8	35,9	42,1	0,83	19,4	20,0
32	MOROCCO	**NASSIRA**	5,3	80,4	43,8	13,2	31,2	36,6	0,74	20,6	20,9
33	MOROCCO	**TOMOUH**	4,4	81,1	39,6	13,9	28,6	34,0	0,76	22,4	22,0
34	SPAIN	**CALERO**	5,4	81,6	39,3	11,9	30,0	35,7	0,73	21,0	20,5
35	SPAIN	**CARPIO**	5,4	82,5	42,8	12,3	33,5	38,5	0,79	19,6	20,3
36	SPAIN	**DON JAIME**	4,7	83,1	49,0	14,4	39,1	36,8	0,75	16,3	20,4
37	SPAIN	**DON PATRICIO**	5,8	82,2	45,3	12,2	33,0	36,5	0,77	19,9	20,7
38	SPAIN	**DON SEBASTIAN**	5,2	83,8	54,6	13,8	36,7	38,2	0,74	16,8	18,9
39	TUNISIA	**KARIM 80**	5,2	82,2	45,7	12,4	30,5	35,2	0,73	20,3	21,0
40	TUNISIA	**MÂALI**	6,1	83,4	57,7	12,0	32,8	35,7	0,73	19,0	20,2
41	TUNISIA	**NASR 99**	4,8	81,5	42,7	12,5	32,0	35,5	0,71	18,7	19,8
42	TUNISIA	**RAZZAK 87**	4,8	82,4	46,6	12,4	31,2	34,5	0,73	19,5	21,0
43	USA-CALIFORNIA	**DESERT KING**	4,4	75,7	32,9	13,0	29,7	38,5	0,84	23,6	20,7
44	USA-CALIFORNIA	**KRONOS**	4,8	80,4	53,9	13,5	31,9	37,7	0,80	21,1	21,1
45	USA-CALIFORNIA	**MOHAWK**	4,1	80,8	53,7	12,7	31,5	36,7	0,71	19,3	19,8
46	USA-CALIFORNIA	**UC1113**	5,5	81,9	42,5	13,0	33,4	37,1	0,77	19,5	20,7

B: Reduced irrigation

Entry	Country of origin	Cross	Grain Yield (t/ha)	Test Weight (TW) kg/Hl	Thousand Kernel Weight (TKW) g	Grain Protein (GPRO) 12.5%MB	Iron Content (FeC) mg/Kg	Zinc Content (ZnC) mg/Kg	Phytic Acid (%)	Molar Ratio Phy:Fe	Molar Ratio Phy:Zn
1	ALGERIA	**KHROUB 76**	2,3	82,6	50,6	15,2	34,6	32,7	0,68	16,4	21,0
2	ALGERIA	**WAHA**	2,9	82,6	40,6	14,4	31,8	28,4	0,64	17,1	22,1
3	ARGENTINA	**BONAERENSE VALVERDE**	2,7	83,2	39,2	13,6	33,4	34,0	0,67	17,0	19,6
4	ARGENTINA	**BUCK CANDISUR**	2,8	82,9	52,0	13,8	32,4	30,5	0,64	17,2	20,4
5	AUSTRALIA	**BELLAROI**	1,5	79,9	48,2	18,2	40,5	43,0	0,92	19,7	21,9
6	AUSTRALIA	**HYPERNO**	3,1	82,6	43,1	13,3	35,7	28,6	0,60	14,2	20,6
7	AUSTRALIA	**JANDAROI**	2,8	82,0	43,0	14,9	34,4	30,8	0,69	17,2	21,4
8	CHILE	**GUAYACAN INIA**	2,4	83,1	48,3	13,8	33,4	31,3	0,63	16,1	19,6
9	SPAIN	**AVISPA**	2,8	83,6	42,2	13,3	33,0	29,1	0,60	15,5	20,1
10	FRANCE	**EXELDUR**	1,1	78,9	36,4	17,3	35,7	44,7	0,84	20,4	18,7
11	INDIA	**HD 4530**	2,5	80,1	39,6	14,6	32,9	30,2	0,52	13,5	17,0
12	INDIA	**MALAVIKA**	2,5	82,9	43,9	12,9	31,9	29,0	0,55	14,6	19,4
13	INDIA	**RAJ 1555**	2,4	82,4	51,5	12,8	31,2	26,6	0,51	13,6	19,0
14	INDIA	**WH 896**	2,6	83,5	45,3	13,1	32,3	32,1	0,56	14,8	17,1
15	ITALIA	**BRONTE**	2,7	81,5	50,8	14,1	34,8	30,6	0,62	15,3	19,1
16	ITALIA	**CRECALE**	2,4	79,6	34,5	14,2	32,4	30,9	0,63	16,8	20,3
17	ITALIA	**DUILLIO**	2,7	82,1	48,3	13,4	33,3	30,4	0,59	15,7	19,5
18	ITALIA	**IRIDE**	2,7	82,7	39,4	13,5	33,2	29,1	0,58	14,9	19,1
19	ITALIA	**KARALIS**	2,5	82,3	46,4	14,6	35,3	35,2	0,68	16,4	18,9
20	ITALIA	**NORMANO**	1,8	82,0	44,3	18,0	38,5	37,2	0,79	17,2	20,7
21	ITALIA	**SARAGOLLA**	2,7	79,8	43,2	13,4	33,8	29,2	0,62	15,6	21,1
22	ITALIA	**SVEVO**	2,7	82,4	40,9	15,7	35,2	32,9	0,65	15,9	19,5
23	MEXICO	**ACONCHI 89**	2,5	83,6	41,3	13,7	33,8	28,2	0,60	15,0	20,7
24	MEXICO	**ALTAR 84**	2,6	84,1	40,4	13,0	33,1	24,8	0,49	12,7	19,8
25	MEXICO	**CEMEXI C 2008**	2,7	83,2	43,1	13,7	32	29,0	0,54	14,3	18,6
26	MEXICO	**NACORI C 97**	2,8	83,2	44,7	13,9	34,0	30,2	0,51	12,5	16,9
27	MEXICO	**RAFI C 97**	2,5	83,1	38,0	13,6	32,7	30,6	0,57	14,8	18,4
28	MEXICO	**RIO COLORADO**	2,7	81,3	48,1	14,1	33,1	29,8	0,59	15,2	19,5

29	MEXICO	**SOOTY_9/RASCON_37**	2,6	83,2	45,6	14,2	32,6	28,7	0,53	13,6	18,6
30	MOROCCO	**AMRIA**	2,6	83,3	46,0	14,9	34,1	29,2	0,62	15,5	20,5
31	MOROCCO	**MARZAK**	2,5	82,7	46,2	15,0	35,5	31,4	0,63	15,2	19,1
32	MOROCCO	**NASSIRA**	2,5	79,9	43,3	14,7	32,6	30,4	0,57	14,9	18,4
33	MOROCCO	**TOMOUH**	2,7	81,5	42,0	14,5	30,2	27,5	0,55	15,3	20,3
34	SPAIN	**CALERO**	2,2	81,5	34,1	13,3	30,3	28,0	0,49	13,6	18,2
35	SPAIN	**CARPIO**	2,8	83,2	43,2	14,0	35,4	33,4	0,62	14,7	17,1
36	SPAIN	**DON JAIME**	2,6	82,8	42,6	13,1	33,9	27,9	0,48	12,1	16,9
37	SPAIN	**DON PATRICIO**	2,9	82,7	41,8	13,5	32,8	31,9	0,56	14,4	17,4
38	SPAIN	**DON SEBASTIAN**	2,8	83,4	53,3	14,6	34,9	32,1	0,57	13,8	17,4
39	TUNISIA	**KARIM 80**	2,4	82,2	45,7	13,6	32,7	30,0	0,60	15,5	19,4
40	TUNISIA	**MÂALI**	3,2	84,4	53,1	13,0	33,9	28,9	0,54	13,5	18,2
41	TUNISIA	**NASR 99**	2,6	82,9	43,6	13,3	31,0	25,7	0,50	14,1	19,9
42	TUNISIA	**RAZZAK 87**	2,8	83,3	51,9	13,9	34,0	30,5	0,59	14,6	18,8
43	USA-CALIFORNIA	**DESERT KING**	1,9	80,0	40,7	16,2	34,5	37,1	0,66	15,9	17,3
44	USA-CALIFORNIA	**KRONOS**	2,6	80,9	51,7	14,4	33,1	32,2	0,60	15,6	19,3
45	USA-CALIFORNIA	**MOHAWK**	2,6	80,1	47,0	14,0	32,5	30,6	0,59	15,2	18,8
46	USA-CALIFORNIA	**UC1113**	3,0	82,0	40,8	13,8	33,4	30,0	0,58	14,7	19,0
